# Geographical variation in the standard physiology of brushtail possums (*Trichosurus*): implications for conservation translocations

**DOI:** 10.1093/conphys/coy042

**Published:** 2018-08-17

**Authors:** Christine E Cooper, Philip C Withers, Suzanne L Munns, Fritz Geiser, William A Buttemer

**Affiliations:** 1 School of Molecular and Life Sciences, Curtin University, Perth, Western Australia, Australia; 2 School of Biological Sciences, University of Western Australia, Perth, Western Australia, Australia; 3 Biomedical Sciences, College of Veterinary and Biomedical Sciences, James Cook University Townsville, Queensland, Australia; 4 Centre for Behavioural and Physiological Ecology, Zoology, University of New England, Armidale, New South Wales, Australia; 5 School of Biological Sciences, University of Wollongong, Wollongong, New South Wales, Australia

**Keywords:** Basal metabolic rate, evaporative water loss, thermal conductance, wildlife management

## Abstract

Identifying spatial patterns in the variation of physiological traits that occur within and between species is a fundamental goal of comparative physiology. There has been a focus on identifying and explaining this variation at broad taxonomic scales, but more recently attention has shifted to examining patterns of intra-specific physiological variation. Here we examine geographic variation in the physiology of brushtail possums (*Trichosurus*), widely distributed Australian marsupials, and discuss how pertinent intra-specific variation may be to conservation physiology. We found significant geographical patterns in metabolism, body temperature, evaporative water loss and relative water economy. These patterns suggest that possums from warmer, drier habitats have more frugal energy and water use and increased capacity for heat loss at high ambient temperatures. Our results are consistent with environmental correlates for broad-scale macro-physiological studies, and most intra-generic and intra-specific studies of marsupials and other mammals. Most translocations of brushtail possums occur into Australia’s arid zone, where the distribution and abundance of possums and other native mammals have declined since European settlement, leading to reintroduction programmes aiming to re-establish functional mammal communities. We suggest that the sub-species *T. vulpecula hypoleucus* from Western Australia would be the most physiologically appropriate for translocation to these arid habitats, having physiological traits most favourable for the extreme *T*_a_, low and variable water availability and low productivity that characterize arid environments. Our findings demonstrate that geographically widespread populations can differ physiologically, and as a consequence some populations are more suitable for translocation to particular habitats than others. Consideration of these differences will likely improve the success and welfare outcomes of translocation, reintroduction and management programmes.

## Teaser

Brushtail possums have a wide geographical distribution. Possums from warmer, drier habitats have more frugal energy and water use and increased heat loss capacity at high temperatures. Consideration of geographical patterns in physiological attributes of source populations will likely improve the success and welfare outcomes of translocations.

## Introduction

A fundamental goal of comparative physiology is to determine how variation in physiological traits and processes differs spatially and/or temporally between and within species (Feder and Block, 1991; Bozinovic *et al.*, 2011). Macro-physiological studies have commonly focused on broad inter-specific comparisons to achieve a wide geographical and taxonomic perspective for environmental or life history influences on a particular variable (Chown *et al.*, 2004; Bozinovic and Naya, 2015). However, this approach generally ignores potential variation within a particular taxonomic group, assuming that the trait(s) in question are fixed for each species being studied (Bozinovic *et al.*, 2009). Variation in physiological traits between spatially separated populations of a single or closely related species has been relatively understudied (Bozinovic and Naya, 2015; McClelland *et al.*, 2016), but recognition of lower-taxonomic-level physiological variation is gaining prominence along with attempts to identify the underlying mechanisms and functional significance when such variability is found (Bozinovic *et al.*, 2009).

Studies of low-level taxonomic variation over a wide spatial scale not only have application to theoretical considerations of physiological function, evolutionary processes, ecological interactions and species assemblages (Chown *et al.*, 2004; Bozinovic *et al.*, 2009; Chown and Gaston, 2016), but they can also address more applied questions concerning impacts of human-induced habitat modification on biodiversity, and of global climate change (Bozinovic *et al.*, 2011). These two key environmental perturbations of the Anthropocene are gaining ever more social, economic and political attention (Feder and Block, 1991; Steffen *et al.*, 2007). We consider here whether there is sufficient spatial variation in the physiology of a widely distributed mammal to warrant consideration in conservation translocation programmes. More broadly, we consider how studies of comparative physiology can enhance the success of wildlife management and ecosystem conservation in a changing environment.

Translocation of species is a widely used conservation tool, but the success rate of translocations is very low worldwide, with only one third to one half of translocation programmes deemed successful. Translocation outcomes in Australia are particularly poor (Griffith *et al.*, 1989; Wolf *et al.*, 1996; Fischer and Lindenmayer, 2000). The appropriateness of the new habitat and characteristics of a translocated species affect the success of these programmes (Sarrazin and Barbault, 1996; Armstrong and Seddon, 2007), and we posit that characterizing the environmental physiology of the animals to be translocated will further improve their chances for survival. Wildlife managers commonly consider factors such as predation, competition, genetics, habitat, reproductive biology, behaviour and disease as influencing the success of conservation measures (Griffith *et al.*, 1989; Sarrrazin and Barbault, 1996; Snyder *et al.*, 1996; Fischer and Lindenmayer, 2000), but basic environmental physiology is generally overlooked (Tarszisz *et al.*, 2014). This is despite the IUCN Guidelines for conservation translocations clearly indicating that the biotic and abiotic habitat needs and basic biology of a species should be known prior to planning a translocation, and specifically recognizing that an assessment of physiological suitability should be made (IUCN/SSC 2013). Physiological assessment can predict a mammal’s likely capacity for survival and reproduction in a particular environment, and therefore evaluation of pertinent physiological variables should improve the success of conservation translocations beyond that achieved with current ecological, behavioural, health and genetic considerations (Tarszisz *et al.*, 2014).

Environmental conditions directly affect an animal’s energy, water and thermal requirements and whether it can successfully survive and reproduce (Wikelski and Cooke, 2006). It is therefore not surprising that there are significant correlations between metabolic, thermal and hygric physiological traits and environmental factors such as temperature and rainfall at a broad inter-specific level for endotherms (e.g. Tieleman and Williams, 2000; Schleucher and Withers, 2001; Lovegrove, 2003; Rezende *et al.*, 2004; Withers *et al.*, 2006; White *et al.*, 2007; van Sant *et al.*, 2012), suggesting that these physiological characteristics are under environmental selection. However, few studies have examined such correlations at lower taxonomic levels, and even fewer describe intra-specific physiological variation, although there is some evidence that geographic variation in physiological traits is consistent with accommodating environmental variables (e.g. Tracy and Walsberg, 2000; Mueller and Diamond, 2001; Tieleman and Williams, 2002; Williams *et al.*, 2004; Careau *et al.*, 2007; Bozinovic *et al.*, 2009; Cooper and Withers, 2010). These studies suggest that it is inappropriate to consider a physiological variable as a fixed species-wide trait, and that intra-specific variation may play an important role in determining the potential survival of individuals translocated to a new geographical location. Translocated individuals must have physiological traits that are appropriate for survival and subsequent reproduction in their new environment, or they must have the phenotypic flexibility to quickly acclimatize. Environmental conditions such as drought have been implicated in the decline of populations of re-introduced species (e.g. Winnard and Coulson, 2008; Facka *et al.*, 2010), thus identifying intra-specific differences among individuals from different geographical locations most likely to physiologically cope with environmental conditions will aid in planning for more successful translocations.

The common brushtail possum (*Trichosurus vulpecula*) is the mostly widely distributed Australian marsupial, with a historical distribution throughout Australia (How, 1983; How and Hillcox, 2000). Despite it being one of the most successful marsupial urban adaptors, the distribution and abundance of the brushtail possum has declined post-European settlement. Declines are particularly prominent in the arid zone where the brushtail possum has disappeared from much of its former range (Kerle *et al.*, 1992; How and Hillcox, 2000). Increasing environmental aridity, compounded by other anthropogenic impacts such as hunting, predation by introduced mammals and land clearing have been implicated in its decline (Kerle *et al.*, 1992). As a consequence of this decline and its wide geographical distribution across the Australian continent, the brushtail possum features in many of the conservation translocation programmes that aim to re-establish functional mammal communities on the Australian mainland, particularly in the arid zone. There are a number of such programmes currently planned or in the early stages of implementation by government and non-government conservation agencies (Short and Hide, 2014; Natural Resource Management South Australia Arid Lands, 2017; Department of Biodiversity, Conservation and Attractions, 2017; Australian Wildlife Conservancy, 2018; Threatened Species Recovery Hub, 2017) and so quantifying the physiological characteristics of brushtail possums on a broad spatial scale, and assessing potential for variation consistent with local environmental conditions, is a timely contribution to these ambitious conservation initiatives.

There is considerable variation in size and colour of brushtail possums, and this has led to the recognition of various sub-species, and in some cases new species, each with distinct geographical ranges (Kerle *et al.*, 1991; Lindenmayer *et al.*, 2002). Body size variation is related to environmental aridity and food availability, with possums from more arid regions having a smaller body size than those from mesic areas (Yom Tov and Nix, 1986). We examine here if this observed anatomical variation is accompanied by physiological variation on a large spatial scale, from south-west Western Australia to north-east Queensland. If pertinent physiological traits are found to vary geographically within this species, then this has important implications for the success of translocation programmes.

## Methods

Brushtail possums were captured in wire cage traps baited with a rolled oat, peanut butter and sardine mixture. Possums were captured and held under licence from the relevant state wildlife authorities, and experiments followed the Australian code of practice for the care and use of animals for scientific purposes, approved by the animal ethics committees of Curtin University, University of Western Australia, James Cook University, University of New England and University of Wollongong.

Six common brushtail possums were caught at Mt Caroline near Kellerberrin, south-west Western Australia (WA, 31.6°S 117.7°E; *Trichosurus vulpecula hypoleucus*), six at Wilton, near Wollongong, New South Wales (NSW, 34.4°S 150.9°E; *Trichosurus vulpecula vulpecula*), and eight were captured near Ayr, northern Queensland (Qld, 19.6°S 147.4°E; *Trichosurus vulpecula johnstonii*). Six short-eared brushtail possums (*Trichosurus caninus*) were captured at Washpool National Park (Northern Tablelands, NSW, 29.3°S 152.3°E). All possums were adult and no females were lactating. The total annual rainfall, mean number of rainy days, and mean minimum and maximum ambient temperatures of the capture locations are presented in Table 1, with the WA site characterized by a warm and dry climate, the two NSW sites cool and moist, and the QLD site being hot and wet (Australian Bureau of Meteorology, 2017). Brushtail possums were held in captivity for several weeks, fed on an *ad libitum* diet of fresh fruit and vegetables, cheese, *Eucalyptus* leaves, small animal muesli and rodent cubes, with *ad libitum* drinking water.

**Table 1:** Climate variables for the capture locations of brushtail possums used in this study

Climate variable	Kellerberrin WA	Wilton NSW	Ayr QLD	Washpool NSW
Mean maximum temperature (°C)	25.2	23.4	29.4	24.9
Mean minimum temperature (°C)	10.8	8.9	17.8	6
Total annual rainfall (mm)	330	805	1060	891
Mean number of rainy days	48	71	52	79

Data from the Australian Bureau of Meteorology (2017).

**Table 2:** Summary of the coefficients for the quadratic equation of a GLMM (with individual as a random factor) of ambient temperate (*T*_a_) on physiological variables (*T*_b_, body temperature; VO_2_, oxygen consumption rate; VCO_2_, carbon dioxide production rate; EWL, evaporative water loss; *C*_wet_, wet thermal conductance; *C*_dry_, dry thermal conductance; RWE, relative water economy) for brushtail possums from Kellerberrin Western Australia (*Trichosurus vulpecula hypoleucus*), Wilton New South Wales (*T. v. vulpecula*), Washpool New South Wales (*T. caninus*) and Ayr Queensland (*T. v. johnstonii*). Individual is included in the GLMM model as a random factor

Variable	Coefficients	*T. v. hypoleucus* for Kellerberrin	*T. v. vulpecula* for Wilton	*T. caninus* for Washpool	*T. v. johnstonii* for Ayr
*T*_b_ (°C)	Intercept	34.5 ± 0.19***	33.4 ± 0.10***	36.0 ± 0.20***	37.8 ± 0.12***
	*T*_a_	4.08 ± 1.19**	4.52 ± 0.39***	4.02 ± 0.47***	6.31 ± 0.60***
	*T*_a_^2^	1.93 ± 1.19^ns^	−0.07 ± 0.39^ns^	1.50 ± 0.46**	2.95 ± 0.61***
VO_2_ (ml O_2_ h^–1^)	Intercept	0.404 ± 0.007***	0.364 ± 0.024***	0.428 ± 0.014***	0.418 ± 0.015***
	*T*_a_	−0.592 ± 0.046***	−0.410 ± 0.036***	−0.395 ± 0.041***	−0.480 ± 0.058***
	*T*_a_^2^	0.346 ± 0.046***	0.209 ± 0.036**	0.140 ± 0.040**	0.374 ± 0.059***
VCO_2_ (ml CO_2_ h^–1^)	Intercept	0.339 ± 0.008***	0.309 ± 0.027**	0.375 ± 0.032***	0.296 ± 0.015***
	*T*_a_	−0.465 ± 0.053***	−0.285 ± 0.084*	−0.342 ± 0.101**	−0.196 ± 0.043***
	*T*_a_^2^	0.226 ± 0.053***	0.137 ± 0.084^ns^	0.242 ± 0.100*	0.224 ± 0.043***
EWL (mg H_2_O h^–1^)	Intercept	0.564 ± 0.049***	0.332 ± 0.022***	0.674 ± 0.044***	0.795 ± 0.055***
	*T*_a_	2.574 ± 0.315***	0.123 ± 0.049^ns^	1.665 ± 0.233***	2.839 ± 0.330***
	*T*_a_^2^	2.380 ± 0.315***	0.109 ± 0.049^ns^	1.278 ± 0.233***	2.740 ± 0.330***
*C*_wet_ (J g^–1^ h^–1^ °C^–1^)	Intercept	1.133 ± 0.131***	0.572 ± 0.041***	1.009 ± 0.049***	1.390 ± 0.115***
	*T*_a_	5.926 ± 0.829***	0.444 ± 0.073***	3.192 ± 0.257***	5.910 ± 0.688***
	*T*_a_^2^	5.184 ± 0.829***	0.197 ± 0.072*	2.043 ± 0.257***	4.569 ± 0.688***
*C*_dry_ (J g^–1^ h^–1^ °C^–1^)	Intercept	0.643 ± 0.048***	0.501 ± 0.039***	0.699 ± 0.045***	1.349 ± 0.111***
	*T*_a_	1.586 ± 0.301***	0.282 ± 0.060**	1.560 ± 0.173***	5.735 ± 0.668***
	*T*_a_^2^	1.008 ± 0.301**	0.163 ± 0.060*	0.668 ± 0.172**	4.434 ± 0.668***
RWE	Intercept	0.766 ± 0.042***	0.720 ± 0.042***	0.545 ± 0.032***	0.481 ± 0.035***
	*T*_a_	−2.412 ± 0.169***	−0.950 ± 0.107***	−1.567 ± 0.160***	−1.474 ± 0.104***
	*T*_a_^2^	−0.437 ± 0.169*	0.184 ± 0.107^ns^	0.072 ± 0.159^ns^	-0.168 ± 0.106^ns^

Values are mean ± standard error. ns, not significant, *P < 0.05, **P < 0.01, ***P < 0.001.

Metabolic rate (MR), as determined by rates of oxygen consumption (VO_2_) and carbon dioxide production (VCO_2_), and evaporative water loss (EWL), were measured using standard flow-through respirometry after Withers (2001). Measurements were made, in random order, at ambient temperatures (*T*_a_) ranging from 6°C to 35°C; not all possums were measured at all *T*_a_ for logistical and ethical reasons. Data for *T. v. hypoleucus* at 26°C and 30°C have been published by Cooper and Withers (2008). Possums were fasted overnight, then measured during their inactive phase (day) for ~8 h, except at *T*_a_ = 34°C where experiments were no longer than 6 h to avoid potential adverse effects of heat exposure and dehydration. Possums were measured at only a single *T*_a_ per day, with at least three days between successive measurements. Body temperature (*T*_b_) was measured using a Radiospares (Smithfield, New South Wales Australia) thermocouple metre (±0.1°C), with a plastic-tipped thermocouple inserted ~2–3 cm into the cloaca immediately after the possum was removed from the chamber at the conclusion of each experiment. Possums were weighed to the nearest gram before and after each experiment, and the mean of the two measures used in calculations.

The respirometry system consisted of an 8000 cm^3^ metabolic chamber placed in a controlled temperature cabinet or room (±~2°C). Airflow through the chamber was achieved via a variety of pumps or a compressed air line; water vapour was removed using Drierite (W.A. Hammond Drierite Co. Ltd, Xenia, OH, USA), and flow rate regulated by a mass flow controller (Aalborg GFC171 or Omega FMAA2412; Orangeburg, NY, USA and Stamford, CT, USA respectively) at 2.5–4.6 l min^–1^ (dependent on *T*_a_ and animal mass). A subsample of excurrent air passed through a Vaisala HMP45A (Helsinki, Finland) relative humidity (RH) and *T*_a_ probe, then through Drierite to carbon dioxide and oxygen analysers (Leybold–Heraeus Binos-C Cologne, Germany, Qubit S153 Kingston, Ontario, or Sable Systems CA-2A Las Vegas, NV, USA CO_2_ analysers; Sable Systems Foxbox or Servomex 572, 574 or OA184 Crowborough, East Sussex, UK, O_2_ analysers). A PC running a custom-written Visual Basic (V6; Microsoft, Redmond, WA, USA) programme recorded the voltage outputs of the analysers every 10–30 s. A baseline of background O_2_, CO_2_ and RH was established for at least 20 min before and after each experiment.

The mass flowmeters were calibrated using a Gilian Gilibrator (Sensidyne, St Petersburg, Florida, USA), traceable to a national standard, or a bubble flowmeter, corrected to standard temperature and pressure dry (STPD). Gas analysers were calibrated using room air (20.95% O_2_), nitrogen (O% O_2_ and CO_2_) and a precision gas mix (0.53% CO_2_, BOC Gases, Perth, Western Australia) and/or a butane flame after Withers (2001). Calibration of the RH probes, achieved by saturating air at a known temperature and then warming to *T*_a_ after Cooper and Withers (2008), was routinely confirmed using 2 points, 1% RH (dried with Drierite) and 100% RH (saturated; by breathing on the probe). A mercury thermometer, traceable to a national standard, was used for temperature calibration.

Respirometry calculations were made after Withers (2001) using a custom-written Visual Basic programme, and resting VO_2_, VCO_2_ and EWL were calculated for a period of at least 20 min during each experiment when values were steady and minimal, indicating the possums were at rest. Respiratory exchange ratio (RER) for each experiment was calculated as VCO_2_/VO_2_, and was used to convert MR to metabolic heat production (MHP) and metabolic water production (MWP) using oxy-calorific and hygric conversion coefficients at the measured RER (Withers *et al.*, 2016). EWL was converted to evaporative heat loss (EHL) using 2.4 J mg^–1^ H_2_O (Withers *et al.* 2016). Wet (*C*_wet_) and dry (*C*_dry_) thermal conductance (J g^–1^ h^–1^ °C^–1^) were calculated as *C*_wet_ = MHP/(*T*_b_–*T*_a_), and *C*_dry_ = (MHP–EHL)/(*T*_b_–*T*_a_). Relative water economy (RWE) was calculated as MWP/EWL and the point of relative water economy (PRWE) as the *T*_a_ where RWE was calculated to be 1.

We used generalized linear mixed effect models (GLMM) to examine *T*_a_ and geographic location effects, while accounting for repeated measurements of individuals as a random factor, using the lmer function in library lme4 (Bates *et al.* 2014) and lmerTest (Kuznetsova *et al.* 2014), in RStudio (RStudio Team, 2015), with Satterthwaite’s approximations for calculation of degrees of freedom. Individual differences between possums were examined with a likelihood ratio test of the random effect. We examined the physiological response to *T*_a_ for each species, with *T*_a_ as a polynomial fixed factor and individual as a random factor. To compare between locations, MR, EWL and *C*_wet_ were expressed as mass-independent values using the marsupial scaling exponents of 0.74, 0.68, 0.57, respectively (Withers *et al.*, 2006). We then compared body mass and physiological responses to *T*_a_ among locations, with *T*_a_ as a polynomial function, location as a fixed factor, and individual as a random factor. Pair-wise location comparisons were made with the most arid habitat sub-species (*T. v. hypoleucus* from WA) as the reference category. Finally, we compared mass-independent standard physiological variables (measured at basal MR; *T*_a_ ~ 26°C) among possums from each location using a one-way ANOVA with Student–Newman–Keuls post-hoc tests, in StatistiXL (v2.1, Nedlands, Western Australia). Values are presented as mean ± standard error (SE), with *N* = number of individuals and *n* = number of measurements.

## Results

Body mass of brushtail possums differed at the various locations (*F*_3,40_ = 5.42, *P* = 0.004); short-eared brushtails (*T. caninus*; 2370 ± 24.8 g, *N* = 6, *n* = 28) were heavier than *T. v. hypoleucus* from WA (1787 ± 22.4 g, *N* = 6, *n* = 41; *t*_30_ = 3.14, *P* = 0.003), and the other sub-species of common brushtail were of intermediate body mass (*T. v. vulpecula* 1992 ± 129.1 g, *N* = 6, *n* = 15; *T. v. johnstonii* 2011 ± 48.1 g, *N* = 8, *n* = 37). There were overall significant differences for individuals within sub-species/species with respect to body mass (*χ*^2^_1_ = 108, *P* < 0.001) and all physiological variables (*χ*^2^_1_ ≥ 5.47, *P* < 0.019).

**Figure 1:**
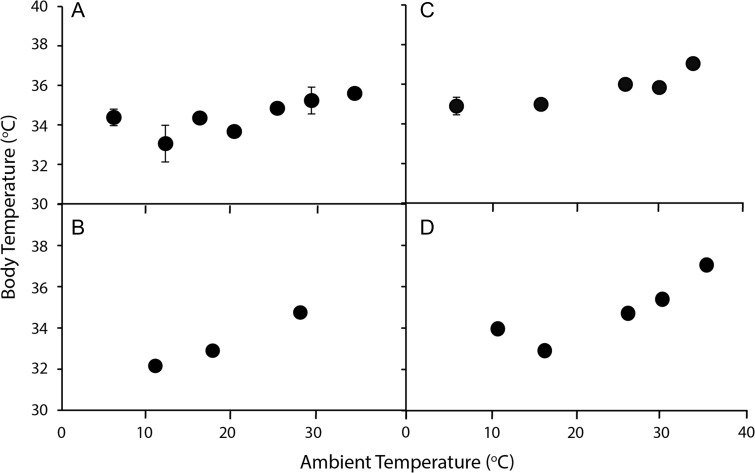
Body temperature of brushtail possums (**A**: *Trichosurus vulpecula hypoleucus*, Western Australia; **B**: *T*. *v*. *vulpecula*, New South Wales; **C**: *Trichosurus caninus*, Tablelands, New South Wales; **D**: *T*. *v*. *johnstonii*, Queensland) at a range of ambient temperatures. Values are mean ± SE, *N* = 6–8.

**Figure 2:**
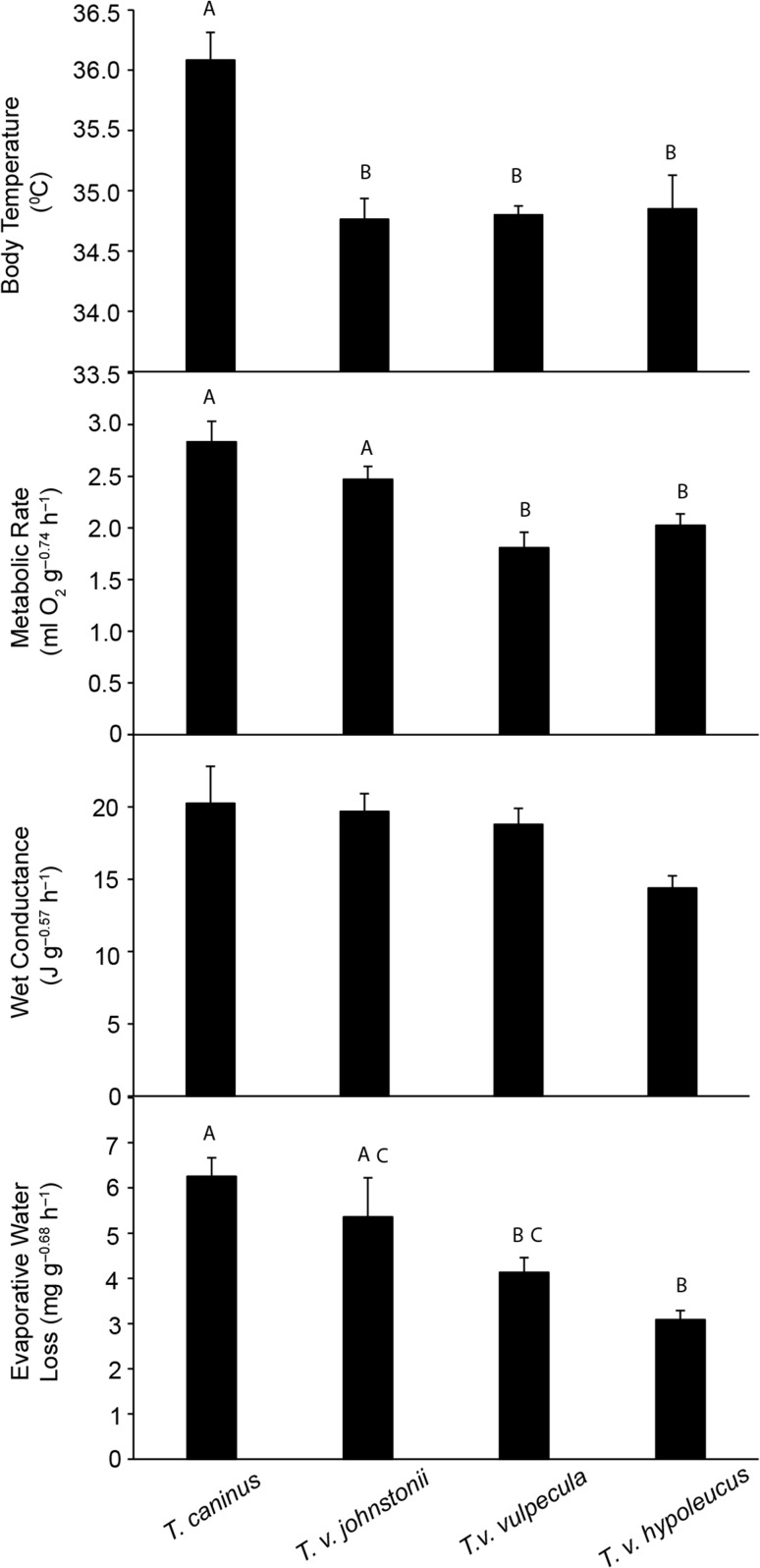
Mass-independent standard physiological variables, measured at an ambient temperature of ~26°C, of brushtail possums from four geographical regions; *Trichosurus caninus* from the northern tablelands of New South Wales (*N* = 6), *T. vulpecula johnstonii* from north Queensland (*N* = 8), *T. v. vulpecula* from New South Wales (*N* = 6) and *T. v. hypoleucus* from Western Australia (*N* = 6). Different letters indicate significant differences at α = 0.05. Values are mean ± SE.

Body temperature of all brushtail possums was positively influenced by *T*_a_ (*F*_1,15–41_ ≥ 7.18, *P* ≤ 0.002; Fig. 1; Table 2). There was a significant location effect (*F*_3,121_ = 11.6, *P* < 0.001) and interaction with *T*_a_ (*F*_3,121_ = 3.68, *P* = 0.014) for the different taxa. Common brushtail possums from WA had a lower *T*_b_ than short-eared brushtails (*T*_121_ = 2.61, *P* = 0.010), but a higher *T*_b_ than the other sub-species of common brushtail (*t*_121_ ≥ 2.26, *P* ≤ 0.028). The influence of *T*_a_ on *T*_b_ was more pronounced for common brushtail possums from NSW and QLD (*t*_121_ ≥ 2.26, *P* ≤ 0.026) than it was for those from WA and for the short-eared brushtail (Fig. 1). Significant differences between the taxa were apparent for standard *T*_b_ (*F*_3,22_ = 13.8, *P* < 0.001; Fig. 2), which was significantly higher (36.2 ± 0.17°C; *N* = 6) for the short-eared brushtail than for common brushtail possums from all locations (34.8–34.9°C; *P* < 0.001; *N* = 6–8).

**Figure 3:**
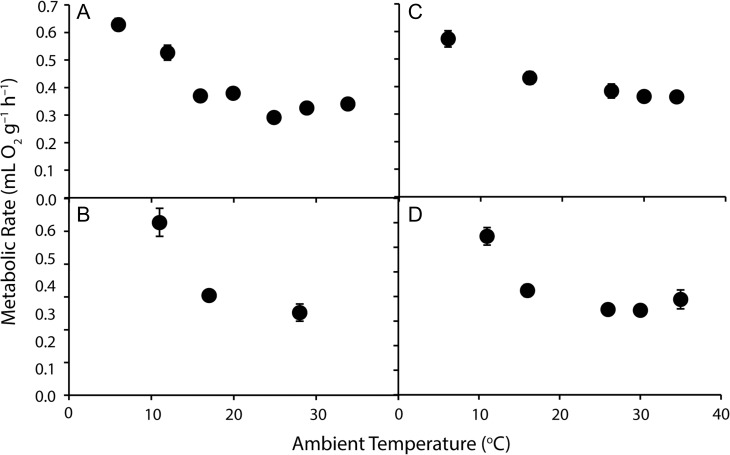
Metabolic rate of brushtail possums (**A**: *Trichosurus vulpecula hypoleucus*, Western Australia; **B**: *T*. *v*. *vulpecula*, New South Wales; **C**: *Trichosurus caninus*, Tablelands, New South Wales; **D**: *T*. *v*. *johnstonii*, Queensland) at a range of ambient temperatures. Values are mean ± SE, *N* = 6–8.

Strong *T*_a_ effects were found for MR (VO_2_) of all brushtail possums (*F*_2,9–41_ ≥ 53.5, *P* ≤ 0.001; Fig. 3; Table 2), with MR decreasing with increasing *T*_a_ to *T*_a_ ~26°C, before stabilizing or increasing at higher *T*_a_. Patterns of VCO_2_ essentially mirrored VO_2_. Brushtail possums from the various locations differed with respect to MR (*F*_3,29_ = 8.3, *P* < 0.001) and there was a significant interaction between *T*_a_ and location (*F*_6,97_ = 7.1, *P* < 0.001). The short-eared brushtail possum and common brushtail from QLD had overall higher MRs (*t*_18_ ≥ 3.34, *P* < 0.004) and a less pronounced response to *T*_a_, than common brushtails from WA, which did not differ from NSW brushtails (*t*_71_ = 0.512, *P* = 0.610). Basal metabolic rate (BMR; at *T*_a_ ~ 26°C) differed between possums from the various locations (*F*_3,22_ = 9.13, *P* < 0.001), with those from WA (2.02 ± 0.112 ml O_2_ g^–0.75^ h^–1^; *N* = 6) and NSW (1.81 ± 0.149 ml O_2_ g^–0.75^ h^–1^; *N* = 6) significantly lower than those from QLD (2.47 ± 0.353 ml O_2_ g^–0.75^ h^–1^, *N* = 8) and the short-eared brushtail (2.83 ± 0.198 ml O_2_ g^–0.75^ h^–1^; *P* < 0.037, *N* = 6; Fig. 2).

**Figure 4:**
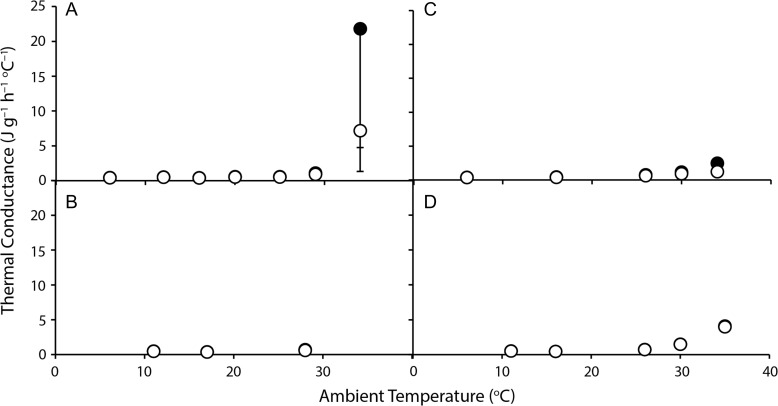
Wet (black symbols) and dry (white symbols) thermal conductance of brushtail possums (**A**: *Trichosurus vulpecula hypoleucus*, Western Australia; **B**: *T. v. vulpecula*, New South Wales; **C**: *Trichosurus caninus*, Tablelands, New South Wales; **D**: *T. v. johnstonii*, Queensland) at a range of ambient temperatures. Values are mean ± SE, *N* = 6–8.

Conductance remained low and relatively constant at *T*_a_ below thermoneutrality, and increased at high *T*_a_ (Fig. 4), *C*_wet_ by 6.1–10.7 times and *C*_dry_ by 3.2–7.4 times at *T*_a_ = 35°C compared to *T*_a_ = 6°C, reflecting the highly significant effects of *T*_a_ for both *C*_wet_ (*F*_2,9–40_ ≥ 21.8, *P* < 0.002) and *C*_dry_ (*F*_2,9–40_ ≥ 41.2, *P* ≤ 0.001; Table 2). There were no overall location (*F*_3,120_ = 1.19, *P* = 0.316), *T*_a_ (*F*_2,120_ = 1.46, *P* = 0.237) or interaction (*F*_6,120_ = 1.82, *P* = 0.010) effects for mass-independent *C*_wet_, and no location differences (*F*_3,21_ = 2.85, *P* < 0.061; Fig. 2) for standard mass-independent *C*_wet_.

**Figure 5:**
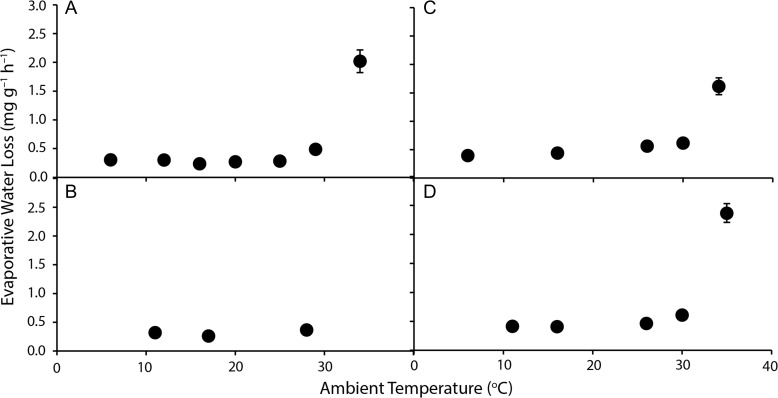
Evaporative water loss of brushtail possums (**A**: *Trichosurus vulpecula hypoleucus*, Western Australia; **B**: *T. v. vulpecula*, New South Wales; **C**: *Trichosurus caninus*, Tablelands, New South Wales; **D**: *T. v. johnstonii*, Queensland) at a range of ambient temperatures. Values are mean ± SE, *N* = 6–8.

**Figure 6:**
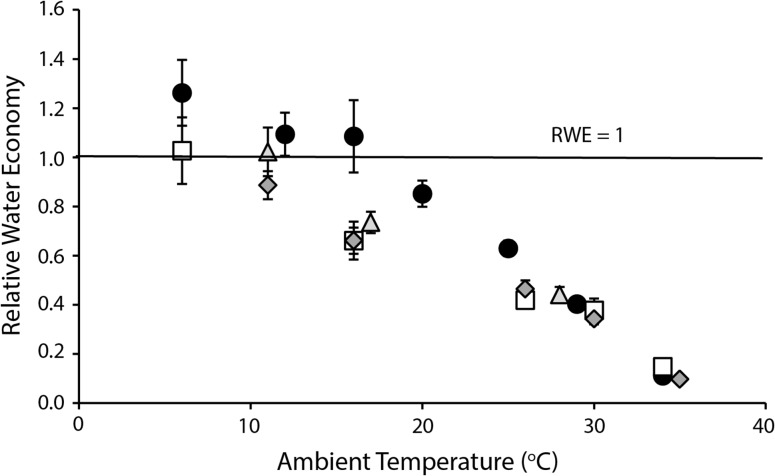
Relative water economy (RWE) of brushtail possums (*Trichosurus vulpecula hypoleucus*, Western Australia, black circles; *T*. *v*. *vulpecula*, New South Wales, light grey triangles; *Trichosurus caninus*, tablelands, New South Wales, white squares; *T. v. johnstonii*, Queensland, dark grey diamonds) at a range of ambient temperatures. Values are mean ± SE, *N* = 6–8.

For all brushtail possums, *T*_a_ affected EWL (*F*_10-41_ ≥ 5.4, *P* < 0.025; Fig. 5; Table 2). EWL was relatively constant at *T*_a_ below thermoneutrality, but increased by 4.3–6.6 times from *T*_a_ ~10°C to 36°C; EHL was 50–73% of MHP at *T*_a_ = 36°C. Location (*F*_3,120_ = 4.9, *P* = 0.003), and its interaction with *T*_a_ (*F*_6,120_ = 5.7, *P* < 0.001), were significant. Brushtail possums from WA differed from those from NSW and QLD (*P* ≤ 0.032), with a lower EWL at low to moderate *T*_a_ and a greater increase at the highest *T*_a_. Standard EWL at *T*_a_ ~26°C was influenced by location (*F*_3,22_ = 5.02, *P* = 0.008), with common brushtail possums from WA having a lower standard EWL (3.09 ± 0.197 mg O_2_ g^–0.68^ h^–1^) than short-eared brushtail possums (6.49 ± 0.412 mg O_2_ g^–0.68^ h^−1^) and common brushtails from QLD (5.36 ± 0.870 mg O_2_ g^–0.68^ h^–1^; Fig. 2). Common brushtail possums from NSW also had lower EWL (4.13 ± 0.805 mg O_2_ g^–0.68^ h^–1^) than the short-eared brushtail possum. The PRWE ranged from 5.9°C (QLD and the short-eared brushtail) to 10.4°C (NSW) and 15.0°C (WA; Fig. 6).

## Discussion

We have quantified significant variation in the physiological traits of spatially separated populations of brushtail possums, marsupials with a wide geographical distribution throughout the Australian continent. This variation was consistent with environmental patterns of inter-specific variation for marsupials (Withers *et al.*, 2006) and other endotherms (e.g. Tieleman and Williams, 2000; Schleucher and Withers, 2001; Lovegrove, 2003; Rezende *et al.*, 2004; White *et al.*, 2007; van Sant *et al.*, 2012), and with anatomical differences identified previously for brushtail possums (Yom Tov and Nix, 1986). We discuss here potential environmental drivers of this geographic variability and then assess the likely impacts of these patterns on management and conservation actions, particularly conservation translocations, for this species and for mammals in general.

Possums from all locations had typical endothermic responses to *T*_a_. Possums were thermoneutral at 26°C, MR increased at lower *T*_a_ while *T*_b_, EWL and C remained relatively constant, and all variables increased at higher *T*_a_. This pattern was consistent with previous observations for *T. v. vulpecula* from NSW (Dawson, 1969; Dawson and Hulbert, 1970), although our measures of BMR and EWL were 16–24% lower, and our standard *T*_b_ was 1.3°C lower (despite almost identical body masses). These differences are presumably due to possums in the earlier studies being restrained and having a thermocouple inserted in the cloaca throughout measurement.

Various macro-physiological studies have reported environmental influences on standard physiological variables that highlight the important role of these factors on the physiological phenotype of mammals. Smaller body mass is well described for many other mammalian species from hot compared to colder habitats (i.e. Bergmann’s rule; reviewed by Meiri and Dayan, 2003). There are a number of environmental correlates with standard physiological variables for marsupials (Withers *et al.* 2006) that are broadly consistent with patterns for mammals (e.g. Lovegrove, 2003; Rezende *et al.*, 2004; Van Sant *et al.*, 2012). Inter-specific environmental correlates for standard marsupial *T*_b_, BMR and EWL indicated that species from arid environments with high rainfall variability have lower values than species from mesic environments with more reliable rainfall.

Broad environmental patterns are also apparent at lower taxonomic levels, although there is a paucity of such studies at broad spatial scales, and some inconsistencies between studies (Bozinovic *et al.*, 2009, 2011). Within genera, species from mesic, cold-climate and high productivity habitats typically have higher BMR and EWL, and lower PRWE, than species from arid, hot and low productivity habitats (Mueller and Diamond, 2001; Williams *et al.*, 2004; Careau *et al.*, 2007; Cooper and Withers, 2010). Broadly, intra-specific physiological differences between populations reflect these intra-generic, and wider inter-specific, patterns for rodents, bats, marsupials and monotremes (Augee, 1978; Tracy and Walsberg, 2000; Geiser and Ferguson, 2001; Bozinovic *et al.*, 2009; Dunbar and Brigham, 2010; Stawski and Geiser, 2011), but there are exceptions. For example, there were no differences in torpor *T*_b_ for northern and southern little red bats (*Lasiurus borealis*), but there were for big brown bats (*Eptesicus fuscus*; Dunbar and Brigham, 2010), while woodrats (*Neotoma* spp.) from different environments had similar MR and *T*_b_ but different *C*_wet_ and body mass (Brown and Lee, 1969). There is currently no clear explanation as to why the established ecological drivers of physiology observed at higher levels of taxonomy are reflected in geographically separated populations of many, but not all, species. Presumably there are a suite of genetic, evolutionary, life history and environmental factors that determine populational differences a over broad geographic scales (Dunbar and Brigham, 2010). A much more comprehensive dataset of broad-scale intra-specific population studies is required to address this question, but it does highlight the requirement for species-specific studies to examine potential geographic effects, when the aim is to inform conservation and management actions.

Our physiological data for brushtail possums are generally consistent with the broad macro-physiological observations and lower-taxonomic level environmental patterns for other mammals. Body mass variation with *T*_a_ is well documented for brushtail possums (Yom Tov and Nix, 1986), consistent with our results. Differences in thermal, metabolic and hygric traits of brushtail possums were also generally consistent with other studies, with possums from warmer/drier habitats having more frugal energy and water use and increased capacity for EHL at *T*_a_ above thermoneutrality. The PRWE, an index of water economy and hence a measure of adaptation to aridity (MacMillen, 1990; MacMillen and Hinds, 1983; Hinds and MacMillen, 1986) provided further evidence that the physiology of *T. v. hypoleucus* from WA is more favourable for drier habitats compared to possums from other environments.

The variation we identify here for brushtail possums throughout their geographic range adds to the growing evidence that basic physiological traits are not necessarily fixed species-specific characteristics, and that local environmental conditions can be significant physiological drivers. Unfortunately, there is little evidence that translocation studies consider this variation in the planning phase. Baker and Gemmell (1999) found that possums translocated from cool-temperature Armidale (NSW) to sub-tropical Brisbane (QLD) had greater immune and hormonal responses, and mortality compared to locally captured possums. Our data, showing significant geographic variation in possum physiology, suggest that climate may have contributed to these results; possums from Armidale likely had a less favourable physiology for the Brisbane climate and this impacted on their health and survival.

Our data do not distinguish between genetic, developmental or acclimatisation differences between possum populations. Acclimation and acclimatization to changing environmental conditions, temperature in particular, have long been well documented for mammals (e.g. Chaffee and Roberts, 1971), and there is also evidence that the environment experienced during development can impact the physiological characteristics of adult mammals (e.g. Riek and Geiser, 2012). Tracey and Walsberg (2001) found that developmental and acclimatory effects on the hygric physiology of kangaroo rats (*Dipodomys merriami*) could be as substantial as genetic differences between populations, and could occur relatively quickly. This suggests that translocated individuals have the potential to adjust to their new environment via phenotypic plasticity, but given the considerable stress experienced by animals prior to, during and after translocation, and their vulnerability immediately post-translocation (Waas *et al.*, 1999; Teixeira *et al.*, 2007; Dickens *et al.*, 2010), it is undesirable to impose the added stressor of a sub-optimal physiology that could reduce an individual’s immediate fitness (e.g. Baker and Gemmell, 1999). In addition, the extreme environmental conditions characteristic of arid habitats are likely to be closer to species’ tolerance limits than more mesic environments (Fuller *et al.*, 2010), so species translocated to these environments from milder habitats may be less able to accommodate their new environment. Appreciating geographic patterns in physiological variation is also likely to improve animal welfare outcomes during translocation programmes. Selection of the most appropriate individuals for translation is recognized as one of the first steps in maximizing animal welfare, including assessing if individuals are suited to their new environment (Harrington *et al.*, 2013).

Despite the potential for acclimatization to account for geographic patterns observed here for brushtail possums, data of Cooper and Withers (2010), which approximated a common garden design for another widespread genus of marsupial (*Dasyurus*), suggests a strong genetic basis to their physiology, reflecting environmental conditions. Even if there are significant genetic differences between brushtail possums populations, with sufficient time, re-introduced populations could respond to a new environment by adaptation (evolution, micro-evolution or epigenetic control of gene expression; Hetem *et al.*, 2014). However, re-introduced populations are by necessity small and may not have the capacity to survive because of the reduced fitness of less-suited individuals, and the variation in the founder population might not provide sufficient adaptive scope.

### Recommendations

Given our observed differences in brushtail possum physiology, together with the findings of Baker and Gemmell (1999) concerning translocation responses of brushtail possums from cool-temperature to sub-tropical environments, we recommend that possums involved in translocation programmes should be sourced from areas with the most similar environmental conditions to the proposed release site, not necessarily possums from the closest geographical location. As it is the arid zone where brushtail possum re-introductions are most desirable (Kerle *et al.*, 1992), then the sub-species *T. v. hypoleucus* would be most physiologically appropriate for these translocations, having physiological traits most favourable for the low productivity, low and variable water availability and extreme *T*_a_ of arid environments.

### Implications for conservation translocations

Similar intra-specific geographic variability in physiological traits for an array of other species (e.g. Tracey and Walsberg, 2000; Mueller and Diamond, 2001; Williams *et al.*, 2004; Bozinovic *et al.*, 2009; Dunbar and Brigham, 2010; Stawski and Geiser, 2011) suggest that our recommendation regarding the physiological suitability for translocation of brushtail possums has general significance to translocation, reintroduction and management programmes for other mammals. Tarszisz *et al.*’s (2014) review of 120 translocation programmes found that only 11 programmes (9%) considered aspects of ‘traditional’ physiology in any way. Even for these, the vast majority involved only clinical health checks or post-release monitoring. Traditional physiological variables are not usually considered as part of initial pre-translocation planning (Tarszisz *et al.*, 2014). Our study of brushtail possums is the first to our knowledge that has assessed basic physiological variation of geographically separated populations of a mammal as a consideration in terms of their suitability for translocation. Our data provide a physiological explanation for previous observations of responses by brushtail possums to translocation (Baker and Gemmel, 1999) and support the suggestion of Cooke *et al.* (2013) that individual variation in physiology and environmental tolerance can potentially impact on long-term translocation success. We conclude from our study that widespread mammals can have considerable geographic variation in basic physiological variables, and that knowledge of location-dependence of thermal, metabolic and hygric physiology is a potentially useful tool in selection of which populations would be most suitable to source individuals for conservation translocations.

## References

[coy042C1] Armstrong DP, Seddon PJ (2007) Directions in reintroduction biology. Trends Ecol Evol 23: 20–25. 18160175 10.1016/j.tree.2007.10.003

[coy042C2] Augee ML (1978) Metabolic consequences of subspecific pelage variations in the echidna. Aust Zool 20: 105–109.

[coy042C3] Australian Bureau of Meteorology (2017) Climate statistics for Australian locations. http://www.bom.gov.au/climate/data/index.shtml (last accessed 16 August 2017).

[coy042C4] Australian Wildlife Conservancy (2018) Wildlife translocations. http://www.australianwildlife.org/field-programs/wildlife-translocations.aspx (last accessed 27 March 2018).

[coy042C5] Baker ML, Gemmell RT (1999) Physiological changes in the brushtail possum (*Trichosurus vulpecula*) following relocation from Armidale to Brisbane, Australia. J Exp Zool 284: 42–49. 10368933 10.1002/(sici)1097-010x(19990615)284:1<42::aid-jez7>3.3.co;2-v

[coy042C6] Bates D, Maechler M, Bolker B, Walker S (2014). lme4: Linear mixed-effects models using Eigen and S4. R package version 1.1–7. http://CRAN.R-project.org/package=lme4

[coy042C7] Bozinovic F, Calosi P, Spicer JI (2011) Physiological correlates of geographic range in animals. Ann Rev Ecol Evol Syst 42: 155–179.

[coy042C8] Bozinovic F, Naya DE (2015) Linking physiology, climate and species distributional ranges In Martin LB, Ghalambor CK, Woods A, eds Chapter 17, Integrative Organismal Biology. John Wiley & Sons, Inc, New York, pp 277–290.

[coy042C9] Bozinovic F, Rojas JM, Broitman BR, Vásquez RA (2009) Basal metabolism is correlated with habitat productivity among populations of degus (*Octodon degus*). Comp Biochem Physiol A 152: 560–564. 10.1016/j.cbpa.2008.12.015 19162212

[coy042C10] Brown JH, Lee AK (1969) Bergmann’s rule and climatic adaptation in woodrats (*Neotoma*). Evol 23: 329–338. 10.1111/j.1558-5646.1969.tb03515.x 28562890

[coy042C11] Careau V, Morand-Ferron J, Thomas D (2007) Basal metabolic rate of canids from hot deserts to cold arctic climates. J Mamm 88: 394–400.

[coy042C12] Chaffee RRJ, Roberts JC (1971) Temperature acclimation in birds and mammals. Annu Rev Physiol 33: 155–202. 4951049 10.1146/annurev.ph.33.030171.001103

[coy042C13] Chown SL, Gaston KJ (2016) Macrophysiology—progress and prospects. Funct Ecol 30: 330–344.

[coy042C14] Chown SL, Gaston KJ, Robinson D (2004) Macrophysiology: large-scale patterns in physiological traits and their ecological implications. Funct Ecol 18: 159–167.

[coy042C15] Cooke SJ, Sack L, Franklin CF, Farrell AP, Beardall J, Wikelski M, Chown SL (2013) What is conservation physiology? Perspectives on an increasingly integrated and essential science. Cons Physiol 1(1): cot001 10.1093/conphys/cot001. PMC4732437 27293585

[coy042C16] Cooper CE, Withers PC (2008) Allometry of evaporative water loss in marsupials: implications of the effect of ambient relative humidity on the physiology of brushtail possums (*Trichosurus vulpecula*). J Exp Biol 211: 2759–2766. 18723532 10.1242/jeb.019463

[coy042C17] Cooper CE, Withers PC (2010) Comparative physiology of Australian quolls (*Dasyurus*; Marsupialia). J Comp Physiol B 180: 857–868. 20217094 10.1007/s00360-010-0452-3

[coy042C18] Dawson TJ (1969) Temperature regulation and evaporative water loss in the brush-tailed possum *Trichosurus vulpecula*. Comp Biochem Physiol 28: 401–407. 5777387 10.1016/0010-406x(69)91353-x

[coy042C19] Dawson TJ, Hulbert AJ (1970) Standard metabolism, body temperature, and surface areas of Australian marsupials. Am J Physiol 218: 1233–1238. 5435424 10.1152/ajplegacy.1970.218.4.1233

[coy042C20] Department of Biodiversity, Conservation and Attractions (2017) Operation Rangelands Restoration A 2020 Management. https://www.dpaw.wa.gov.au/about-us/science-and-research/animal-conservation-research/260-rangelands-restoration-reintroduction-of-native-mammals-to-lorna-glen (last accessed 30 October 2017).

[coy042C21] Dickens MJ, Delehanty DJ, Romero ML (2010) Stress: an inevitable component of animal translocation. Biol Conserv 143: 1329–1341.

[coy042C22] Dunbar MB, Brigham RM (2010) Thermoregulatory variation among populations of bats along a latitudinal gradient. J Comp Physiol B 180: 885–893. 20213177 10.1007/s00360-010-0457-y

[coy042C23] Facka AN, Romer GW, Mathis VL, Kam M, Geffen E (2010) Drought leads to collapse of black-tailed prairie dog populations reintroduced to the Chihuahuan desert. J Wild Manag 74: 1752–1762.

[coy042C24] Feder ME, Block BA (1991) On the future of animal physiological ecology. Funct Ecol 5: 136–144.

[coy042C25] Fischer J, Lindenmayer DB (2000) An assessment of the published results of animal relocations. Biol Cons 96: 1–11.

[coy042C26] Fuller A, Dawson T, Helmuth B, Hetem RS, Mitchell D, Maloney SK (2010) Physiological mechanisms in coping with climate change. Physiol Biochem Zool 83: 713–720. 20578846 10.1086/652242

[coy042C27] Geiser F, Ferguson C (2001) Intraspecific differences in behaviour and physiology: effects of captive breeding on patterns of torpor in feathertail gliders. J Comp Physiol B 171: 569–576. 11686615 10.1007/s003600100207

[coy042C28] Griffith B, Scott MJ, Carpenter JW, Reed C (1989) Translocation as a species conservation tool: status and strategy. Science 245: 477–480. 17750257 10.1126/science.245.4917.477

[coy042C29] Harrington LA, Moehrenschlager A, Gelling M, Atkinson RPD, Huges J, Macdonald DW (2013) Conflicting and complementary ethics of animal welfare considerations in reintroductions. Cons Biol 27: 486–500. 10.1111/cobi.12021 23506045

[coy042C30] Hetem RS, Fuller A, Maloney SK, Mitchell D (2014) Responses of large mammals to climate change. Temperature 1: 115–127. 10.4161/temp.29651 PMC4977165 27583293

[coy042C31] Hinds DS, MacMillen RE (1986) Scaling of evaporative water loss in marsupials. Physiol Zool 59: 1–9.

[coy042C32] How RA (1983) Common brushtail possum In Strahan R, ed Complete Book of Australian Mammals. Angus and Robertson, Sydney, pp 147–148.

[coy042C33] How RA, Hillcox SJ (2000) Brushtail possum, *Trichosurus vulpecula*, populations in south-western Australia: demography, diet and conservation status. Wild Res 27: 81–89.

[coy042C34] IUCN/SSC (2013) *Guidelines for Reintroductions and Other Conservation Translocations*, Version 1.0. IUCN Species Survival Commission, Gland, Switzerland, pp viii + 57 pp.

[coy042C35] Kerle JA, Foulkes JN, Kimber RG, Papenfus D (1992) The decline of the brushtail possum, *Trichosurus vulpecula* (Kerr 1798), in arid Australia. Range J 14: 107–127.

[coy042C36] Kerle JA, Mckay GM, Sharman GB (1991) A systematic analysis of the brushtail possum, *Trichosurus vulpecula* (Kerr, 1792) (Marsupialia, Phalangeridae). Aust J Zool 39: 313–331.

[coy042C37] Kuznetsova A, Brockhoff PB, Christensen RHB (2014) lmerTest: Tests in Linear Mixed Effects Models. R package version 2.0–20. http://CRAN.R-project.org/package=lmerTest

[coy042C38] Lindenmayer DB, Dubach J, Viggers KL (2002) Geographic dimorphism in the mountain brushtail possum (*Trichosurus caninus*): the case for a new species. Aust J Zool 50: 369–393.

[coy042C39] Lovegrove BG (2003) The influence of climate on the basal metabolic rate of small mammals: a slow-fast metabolic continuum. J Comp Physiol B 173: 87–112. 12624648 10.1007/s00360-002-0309-5

[coy042C40] MacMillen RE (1990) Water economy of granivorous birds: a predictive model. Condor 92: 379–392.

[coy042C41] MacMillen RE, Hinds DS (1983) Water regulatory efficiency in heteromyid rodents: a model and its application. Ecology 64: 152–164.

[coy042C42] McClelland GTW, McKechnie AE, Chown SL (2016) Basal metabolic rate of the black-faced sheathbill (*Chionis minor*): Intraspecific variation in a phylogenetically distinct island endemic. Physiol Biochem Zool 89: 141–150. 27082724 10.1086/685411

[coy042C43] Meiri S, Dayan T (2003) On the validity of Bergmann’s Rule. J Biogeog 30: 331–351.

[coy042C44] Mueller P, Diamond J (2001) Metabolic rate and environmental productivity: well-provisioned animals evolved to run and idle fast. Proc Nat Acad Sci USA 98: 12550–12554. 11606744 10.1073/pnas.221456698 PMC60091

[coy042C45] Natural Resource Management South Australia Arid Lands (2017) http://www.naturalresources.sa.gov.au/aridlands/plants-and-animals/native-plants-and-animals/bounceback/western-quoll-idnya (last accessed 30 October 2017).

[coy042C46] Rezende EL, Bozinovic F, Garland T (2004) Climatic adaptation and the evolution of maximum and basal rates of metabolism in rodents. Evolution 58: 1361–1374. 15266984 10.1111/j.0014-3820.2004.tb01714.x

[coy042C47] Riek A, Geiser F (2012) Developmental phenotypic plasticity in a marsupial. J Exp Biol 215: 1552–1558. 22496292 10.1242/jeb.069559

[coy042C48] RStudio Team (2015). RStudio: Integrated Development for R. RStudio, Inc., Boston, MA. http://www.rstudio.com/

[coy042C49] Sarrazin F, Barbault R (1996) Reintroduction: challenges and lessons for basic ecology. TREE 11: 474–478. 21237930 10.1016/0169-5347(96)20092-8

[coy042C50] Schleucher E, Withers PC (2001) Re-evaluation of the allometry of wet thermal conductance for birds. Comp Biochem Physiol A 129: 821–827. 10.1016/s1095-6433(01)00356-7 11440868

[coy042C51] Short J, Hide A (2014) Successful reintroduction of the brushtail possum to Wadderin Sanctuary in the eastern wheatbelt of Western Australia. Aust Mamm 36: 229–241.

[coy042C52] Snyder NFR, Derrickson SR, Beissinger SR, Wiley JW, Smith TB, Toone WD, Miller B (1996) Limitations of captive breeding in endangered species recovery. Cons Biol 10: 338–348.

[coy042C53] Stawski C, Geiser F (2011) Do season and distribution affect thermal energetics of a hibernating bat endemic to the tropics and subtropics? Am J Physiol 301: R542–R547. 10.1152/ajpregu.00792.2010 21632847

[coy042C54] Steffen W, Crutzen PJ, McNeill JR (2007) The Anthropocene: are humans now overwhelming the great forces of nature? Ambio 36: 614–621. 18240674 10.1579/0044-7447(2007)36[614:taahno]2.0.co;2

[coy042C55] Tarszisz E, Dickman CR, Munn AJ (2014) Physiology in conservation translocations. Cons Physiol 2: 1–19. 10.1093/conphys/cou054 PMC4732500 27293675

[coy042C56] Teixeira CP, DeAevedo CS, Mendl M, Cipreste CF, Young RJ (2007) Revisiting translocation and reintroduction programmes: the importance of considering stress. Anim Behav 73: 1–13.

[coy042C57] Threatened Species Recovery Hub (2017) http://www.nespthreatenedspecies.edu.au/projects/learning-from-translocation (last accessed 30th October 2017).

[coy042C58] Tieleman BI, Williams JB (2000) The adjustment of avian metabolic rates and water fluxes to desert environments. Physiol Biochem Zool 73: 461–479. 11009400 10.1086/317740

[coy042C59] Tieleman BI, Williams JB (2002) Cutaneous and respiratory water loss in larks from arid and mesic environments. Physiol Biochem Zool 75: 590–599. 12601615 10.1086/344491

[coy042C60] Tracey RL, Walsberg GE (2000) Prevalence of cutaneous evaporation in Merriam’s kangaroo rat and its adaptive variation at the subspecific level. J Exp Biol 203: 773–781. 10648219 10.1242/jeb.203.4.773

[coy042C61] Tracey RL, Walsberg GE (2001) Developmental and acclimatory contributions to water loss in a desert rodent: Investigating the time course of adaptive change. J Comp Physiol B 171: 669–679. 11765976 10.1007/s003600100218

[coy042C62] Van Sant MJ, Oufiero CE, Muñoz-Garcia A, Hammond KA, Williams JB (2012) A phylogenetic approach to total evaporative water loss in mammals. Physiol Biochem Zool 85: 526–532. 22902381 10.1086/667579

[coy042C63] Waas JR, Ingram JR, Matthews LR (1999) Real-time physiological responses of red deer to translocations. J Wild Manag 63: 1152–1162.

[coy042C64] White CR, Blackburn TM, Martin GR, Butler PJ (2007) Basal metabolic rate of birds is associated with habitat temperature and precipitation, not primary productivity. Proc Royal Soc Lond B 274: 287–293. 10.1098/rspb.2006.3727 PMC1685844 17148258

[coy042C65] Wikelski M, Cooke SJ (2006) Conservation physiology. TREE 21: 38–46. 16701468 10.1016/j.tree.2005.10.018

[coy042C66] Williams JB, Muńoz-Garcia A, Ostrowski S, Tieleman BI (2004) A phylogenetic analysis of basal metabolism, total evaporative water loss, and life-history among foxes from desert and mesic regions. J Comp Physiol B 174: 29–39. 14564467 10.1007/s00360-003-0386-0

[coy042C67] Winnard AL, Coulson G (2008) Sixteen years of Eastern Barred Bandicoot *Perameles gunnii* reintroductions in Victoria: a review. Pacific Cons Biol 14: 34–53.

[coy042C68] Withers PC (2001) Design, calibration and calculation for flow-through respirometry systems. Aust J Zool 49: 445–461.

[coy042C69] Withers PC, Cooper CE, Larcombe A (2006) Environmental correlates of physiological variables in marsupials. Physiol Biochem Zool 79: 437–453. 16691511 10.1086/501063

[coy042C70] Withers PC, Cooper CE, Maloney SK, Bozinovic F, Cruz-Neto AP (2016) Ecological and Environmental Physiology of Mammals. Oxford University Press, Oxford, UK.

[coy042C71] Wolf CM, Griffith B, Reed C, Temple SA (1996) Avian and mammalian translocations: update and reanalysis of 1987 survey data. Cons Biol 10: 1142–1154.

[coy042C72] Yom Tov Y, Nix H (1986) Climatological correlates for body size of five Australian mammals. Biol J Linn Soc 29: 245–262.

